# The relationship between pre-operative right ventricular longitudinal strain and low-cardiac-output syndrome after surgical aortic valve replacement

**DOI:** 10.3389/fcvm.2023.1067984

**Published:** 2023-01-19

**Authors:** Yong-jian Zhang, Hong Chen, Ya-ling Dong, Jia-nan Shang, Li-tao Ruan, Yang Yan, Yan Song

**Affiliations:** ^1^Department of Cardiovascular Surgery, The First Affiliated Hospital of Xi’an Jiaotong University, Xi’an, Shaanxi, China; ^2^Department of Ultrasound, The First Affiliated Hospital of Xi’an Jiaotong University, Xi’an, Shaanxi, China

**Keywords:** low cardiac output syndrome, right ventricular free wall longitudinal strain, right ventricular function, aortic valve replacement, echocardiography

## Abstract

**Objectives:**

This study was performed to investigate the relationship between right ventricular free wall longitudinal strain (RVFWSL) and low cardiac output syndrome (LCOS) after surgical aortic valve replacement (SAVR) and to further explore its association with readmission within 2 years in patients who developed LCOS after SAVR.

**Methods:**

This single-center retrospective observational study involved consecutive patients who underwent SAVR at our hospital from May 2018 to June 2020. Preoperative echocardiography was obtained within 3 days before SAVR. The longitudinal strain of the right ventricle was analyzed using the right ventricle as the main section, and the RVFWSL and right ventricular four-chamber longitudinal strain (RV4CSL) were obtained. The primary observation was the occurrence of LCOS. The secondary prognostic indicators were mainly the readmission rates within 2 years.

**Results:**

In total, 146 patients were finally included in this study. The RVFWSL was significantly lower in the LCOS group than in the No-LCOS group (16.63 ± 2.10) vs. (23.95 ± 6.33), respectively; *P* < 0.001). The multivariate regression analysis showed that the RVFWSL was associated with LCOS (odds ratio, 1.676; 95% confidence interval, 1.258–2.232; *P* < 0.001). The receiver operating characteristic curve showed that the cut-off value for RVFWSL to predict LCOS was less than –18.3, with an area under the curve of 0.879, sensitivity of 100%, and specificity of 80.47%. The multivariate regression analysis showed that LCOS was an independent risk factor for readmission within 2 years in patients undergoing SAVR.

**Conclusion:**

Patients with RVFWSL (<-18.3%) may be an increased risker for LCOS after SAVR. The occurrence of LCOS after SAVR is Yong-jian Zhang a risk factor for readmission within 2 years. Right ventricular function monitoring may have some predictive value for the postoperative prognosis in patients undergoing SAVR.

## 1. Introduction

Aortic valve disease is the most common heart valve disease (1, 2). Surgical aortic valve replacement (SAVR) remains the predominant treatment for aortic valve disease. With advances in surgical techniques, mortality associated with cardiac surgery has substantially declined; however, the average perioperative mortality rate is currently 1–2%, and the incidence of major postoperative cardiovascular complications remains high (3, 4). Low cardiac output syndrome (LCOS) is the most serious complication after cardiac surgery, and the LCOS is strongly associated with short- and long-term postoperative mortality (5). Previous studies have revealed a mortality rate of more than 20% in patients who develop LCOS after cardiac surgery (6). It is important to rapidly and accurately identify LCOS after surgery. However, it is equally important to be able to accurately predict the occurrence of LCOS in patients before surgery to help the surgeon choose the most appropriate procedure and to reduce the time taken to determine LCOS postoperatively.

Studies have suggested that right ventricular (RV) function is strongly associated with a poor prognosis in patients with aortic valve disease after surgery (7–9). Recent studies have also concluded that RV function parameters such as the RV fractional area change (RVFAC) and tissue Doppler imaging-derived systolic velocity (TDI s’) are strongly associated with cardiovascular mortality 3 years after cardiac surgery (10). Strain imaging has been considered a very important risk assessment tool in recent years; it is highly reproducible and has better predictive power than traditional echocardiographic parameters (11, 12). It has also been suggested that RV longitudinal strain is associated with prognosis in patients with different cardiac diseases (13, 14). However, the predictive value of RV longitudinal strain in relation to postoperative LCOS in patients with aortic valve disease has been less thoroughly reported. Therefore, the aim of this study was to investigate the relationship between RV free wall longitudinal strain (RVFWSL) and LCOS after surgical treatment of aortic valve disease and to further explore its association with readmission within 2 years in patients who developed LCOS after SAVR.

## 2. Materials and methods

### 2.1. Study population

**FIGURE 1:**
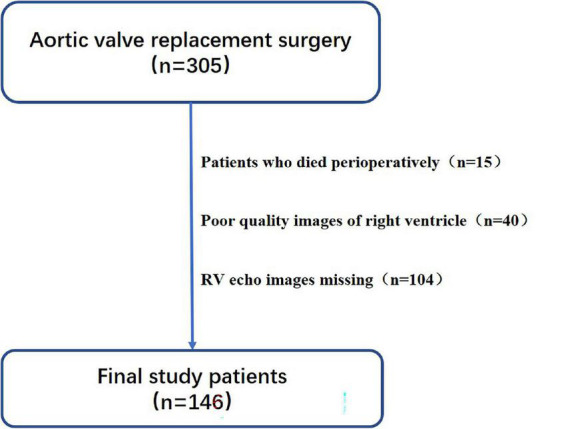
Patient selection and study design.

This single-center retrospective observational study involved consecutive patients who underwent SAVR at our hospital from May 2018 to June 2020. Patients undergoing SAVR included those with aortic stenosis and aortic regurgitation; we referred to the latest guidelines for the diagnostic criteria (1, 2). The inclusion criteria were an age of >18 years and the presence of aortic valve disease. The exclusion criteria were perioperative death, poor-quality images of the right ventricle, and missing RV echo images. Poor quality was considered if the entire annulus of the tricuspid valve and the apex were not well visualized in the apical four-chamber view during the complete cardiac cycle or if foreshortening of the ventricle was present (15). The present study complied with the Declaration of Helsinki and was approved by the local ethics committee of the authors’ institution. All patients provided written informed consent. Figure 1 displays the patient selection and study design.

### 2.2. Echocardiography

A color Doppler ultrasound diagnostic instrument (Philips CX50; Philips Medical Devices Group, Netherlands) equipped with a 1- to 5-MHz cardiac probe was used. Scans were performed and analyzed by two researchers with 5 years of experience in echocardiography. Preoperative echocardiography was obtained within 3 days before SAVR. Postoperative echocardiography was obtained before 10:00 on the first and second day after SAVR. Conventional views were obtained according to the American Society of Echocardiography guidelines, and measurements were performed as recommended by current guidelines (16). We measured overall left ventricular (LV) systolic function using the biplane Simpson method according to the American Society of Echocardiography guidelines (16). LV systolic function was divided into four grades for men (52–72%, normal range; 41–51%, mild abnormality; 30–40%, moderate abnormality; and <30%, severe abnormality) and women (54–74%, normal range; 41–53%, mild abnormality; 30–40%, moderate abnormality; and <30%, severe abnormality) (16).

**FIGURE 2:**
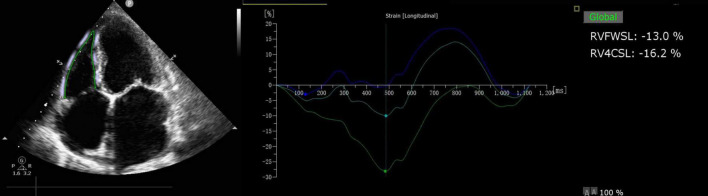
RVFWSL and RV4CSL analysis. Offline analysis was performed using QLAB 10.3 software (cardiac motion quantification; Phillips Medical Systems) to analyze the longitudinal myocardial strain in the right ventricle. The longitudinal strain of the right ventricle was analyzed using the right ventricle as the main section, and the RVFWSL and the RV4CSL were obtained.

Offline analysis was performed using QLAB 10.3 software (cardiac motion quantification; Phillips Medical Systems) to analyze the longitudinal myocardial strain in the right ventricle. The longitudinal strain of the right ventricle was analyzed using the right ventricle as the main section, and the RVFWSL and RV four-chamber longitudinal strain (RV4CSL) were obtained as shown in Figure 2.

### 2.3. Outcomes

The primary observation was the occurrence of LCOS. The criteria for the diagnosis of LCOS were the need for positive inotropic drugs (dobutamine, levosimendan, epinephrine, or milrinone) for at least 12 h after admission to the intensive care unit postoperatively and one of the following within 12 h of transfer to the intensive care unit: pulmonary capillary wedge pressure of >18 mmHg, central venous oxygen saturation of <60%, or urine output of <0.5 mL/kg/h (17).

The secondary prognostic indicators were mainly the readmission rates within 2 years, including readmission within 30 days of the patient’s initial admission. The primary cause of readmission was cardiac in origin.

### 2.4. Statistical analysis

Data analyses were performed with IBM SPSS Statistics for Windows, Version 20.0 (IBM Corp., Armonk, NY, USA). Data are expressed as mean ± standard deviation, median and interquartile range (25th and 75th percentiles), or frequency (%). Categorical variables are expressed as absolute numbers with percentages. Parameters in the LCOS and No-LCOS groups were compared using the chi-squared test or Fisher’s exact test for categorical variables and the unpaired t-test or Mann–Whitney U test for continuous variables as appropriate. For variables with a statistically significant difference, a univariate logistic regression was performed to predict the event. After the univariate analysis, a multivariate logistic regression was performed to identify the independent predictors. To explore the optimal cutoff value of variable to predict the event, a receiver operating characteristic curve was constructed. Interobserver and intraobserver variability for RVFWSL were assessed in 20 patients using the intraclass correlation coefficient (ICC) (15). A *P* value of <0.05 was considered statistically significant.

## 3. Results

### 3.1. Baseline characteristics of patients with and without LCOS

After excluding patients with poor right heart images, 146 patients were finally included in this study. The mean age of all patients was 56 years (range, 49–79 years), and 106 (72.60%) patients were male and 40 (27.40%) were female. Of all 146 patients, LCOS occurred in 18 (incidence of approximately 13.33%). All 18 patients with LCOS needed positive inotropic drugs (dobutamine or epinephrine) at least 12 h after admission to the intensive care unit postoperatively and central venous oxygen saturation of <60% within 12 hours of transfer to the intensive care unit. All patients were divided into an LCOS group and No-LCOS group according to whether they had developed LCOS after surgery (Table 1).

**TABLE 1:** Baseline characteristics in patients with LCOS.

Variable	LCOS (*n* = 18)	No-LCOS (*n* = 128)	*P*
Age (y)	57.5 (49.0–66.25)	56.0 (48.25–62.0)	0.549
Male (*n*, %)	10 (55.6)	96 (75.0)	0.083
Hyapertension (*n*, %)	8 (44.4)	46 (35.9)	0.484
Diabetes mellitus (*n*, %)	1 (5.6)	3 (2.3)	0.434
Chronic kidney disease (*n*, %)	0 (0)	1 (0.8)	0.707
Atrial fibrillation (*n*, %)	1 (5.6)	6 (4.7)	0.872
Stroke (*n*, %)	1 (5.6)	13 (10.2)	0.535
NYHA class (*n*, %)			0.856
I	1 (5.6)	1 (0.8)	–
II	1 (5.6)	13 (10.2)	–
III	14 (77.8)	102 (79.7)	–
IV	2 (11.1)	12 (9.4)	–
Medicine history			
Antiplatelet agents (*n*, %)	1 (5.6)	14 (10.9)	0.481
RAS blockers (*n*, %)	0 (0)	19 (14.8)	0.080
Beta blockers (*n*, %)	4 (22.2)	36 (28.1)	0.599
Aldosterone receptor blockers (*n*, %)	2 (11.1)	14 (10.9)	0.982
Diuretics (*n*, %)	3 (16.7)	37 (28.9)	0.276
Digoxin (*n*, %)	2 (11.1)	14 (10.9)	0.982
Amiodarone (*n*, %)	0 (0)	6 (4.7)	0.348
SBP (mmHg)	121.0 (109.75–149.75)	122.0 (115.0–139.7)	0.752
DBP (mmHg)	71.94 ± 9.86	70.27–12.37	0.584
Mitral valve replacement (*n*, %)	8 (44.4)	55 (43.0)	0.906
Mitral valvuloplasty (*n*, %)	4 (22.2)	10 (7.8)	0.052
Tricuspid valvuloplasty (*n*, %)	8 (44.4)	43 (33.6)	0.366
Cardiopulmonary bypass time (min)	122.50 (95.50–139.50)	109.0 (91.25–134.0)	0.439
Cross-clamp time (min)	83.50 (71.75–95.25)	84.5 (65.0–104.0)	0.983
Haemoglobin (g/L)	129.0 (124.50–143.75)	138.0 (121.25–149.75)	0.327
Erythrocyte pressure (%)	39.35 (37.73–42.28)	41.85 (37.40–44.86)	0.204
Platelets (×10^9^/L)	180.0 (152.25–224.0)	168.0 (134.0–208.5)	0.411
Total protein (g/L)	64.19 ± 6.73	63.90 ± 6.33	0.856
Total cholesterol (mmol/L)	3.55 ± 0.38	3.58 ± 0.85	0.927
Lactate dehydrogenase (U/L)	232.0 (193.0–298.0)	231.0 (199.75–283.50)	0.994
Hydroxybutyrate dehydrogenase (U/L)	194.0 (165.50–231.0)	191.0 (163.75–235.25)	0.929
Creatine kinase (U/L)	42.0 (37.0–65.0)	67.50 (45.75–92.25)	0.049
Creatine kinase isoenzyme (U/L)	11.0 (9.50–16.0)	14.0 (11.0–17.0)	0.113
Hospitalization days in intensive care unit	4.50 (3.0–5.0)	3.0 (2.0–4.0)	0.005
Total hospitalization days	17.0 (15.75–22.25)	15.0 (13.0–19.0)	0.026

LCOS, low-cardiac-output syndrome; SBP, systolic blood pressure; DBP, diastolic blood pressure.

The hospitalization days in the intensive care unit and total hospitalization days of patients with LCOS were significantly higher than those of patients with No-LCOS, which was statistically significant (Table 1).

RVFWSL showed good reproducibility with an intra-observer variability ICC of 0.906 (95% confidence interval, 0.780–0.962; *P* < 0.001) and an inter-observer variability ICC of 0.893 (95% confidence interval, 0.748–0.956; *P* < 0.001).

The comparison of LV-related echocardiographic parameters between the two groups showed no statistically significant differences in the LV end-diastolic diameter, LV end-systolic diameter, or LV ejection fraction (LVEF) between the two groups. The LVEF on the first and second postoperative day in the LCOS group were lower than those in the No-LCOS group, and the difference between the groups was statistically significant (Table 2).

**TABLE 2:** Echocardiographic parameters in patients with LCOS.

Variable	LCOS (*n* = 18)	No-LCOS (*n* = 128)	*P*
LVEDD (mm)	66.5 (49.25–75.75)	60.0 (55.0–69.0)	0.607
LVESD (mm)	45.50 (32.25–56.0)	40.0 (37.0–51.0)	0.631
Interventricular septum (mm)	9.0 (8.0–10.5)	9.0 (8.0–11.0)	0.849
Posterior wall (mm)	9.0 (9.0–10.25)	9.0 (8.0–10.0)	0.531
LVEF (%)	56.50 (45.50–64.50)	60.0 (50.0–64.75)	0.623
LVEF grades			0.552
Normal	11 (61.11)	92 (71.87)	–
Mild abnormality	4 (22.22)	20 (15.63)	–
Moderate abnormality	3 (16.67)	12 (9.38)	–
Severe abnormality	0	4 (3.12)	–
Aortic stenosis ≥ moderate (*n*, %) (*n*, %)	6 (33.3)	39 (30.5)	0.805
Aortic regurgitation ≥ moderate (*n*, %)	12 (66.7)	98 (76.6)	0.362
D_RVB_ (mm)	38.98 ± 5.66	37.66 ± 7.35	0.467
D_RVM_ (mm)	29.78 ± 4.09	27.97 ± 6.27	0.236
TR pressure gradient	26.5 (9.75–42.5)	36.60 (19.85–52.6)	0.893
SysPAP	18.50 (13.0–34.75)	28.60 (23.1–44.85)	0.893
TAPSE/SysPAP ratio	0.48 (0.29–0.92)	0.65 (0.42–0.96)	0.296
RV-FAC (%)	9.62 ± 2.36	11.29 ± 2.21	0.022
TDI s’ (cm/s)	8.95 (8.28–10.38)	11.29 (9.50–12.63)	0.002
TAPSE (mm)	18.0 (15.0–20.0)	20.50 (17.0–23.0)	0.021
RVFWSL (%)	–(16.63 ± 2.10)	–(23.95 ± 6.33)	<0.001
RV4CSL (%)	–(16.79 ± 4.43)	–(19.86 ± 4.99)	0.015
LVEF on the 1st postoperative day (%)	45.0 (33.75–53.25)	52.0 (42.0–58.0)	0.015
LVEF on the 2nd postoperative day (%)	41.50 (32.50–55.25)	55.0 (42.0–60.0)	0.015

LCOS, low-cardiac-output syndrome; LV, left ventricular; LVEDD, LV end-diastolic diameter; LVESD, LV end-systolic diameter; LVEF, left ventricular ejection fraction; D_RVB_, diameter of right ventricular baseline, D_RVM_, diameter of right ventricular mid-section; RV-FAC, right ventricular fractional area change; TDI, tissue Doppler imaging; TAPSE, tricuspid annular plane systolic excursion; RVFWSL, right ventricle free wall longitudinal strain; RV4CSL, right ventricle four-chamber longitudinal strain.

The comparison of RV-related parameters between the two groups showed that the differences in diameter of right ventricular baseline (D_RVB_) and diameter of right ventricular mid-section (D_RVM_) between the two groups were not statistically significant (Table 2).

The RVFAC, TDI s’, and tricuspid annular plane systolic excursion (TAPSE), which are used to assess overall RV function, were significantly lower in the LCOS group than in the No-LCOS group (*P* < 0.05 for all) (Table 2).

The RV strain parameters RVFWSL (16.63 ± 2.10 vs. 23.29 ± 5.37) and RV4CSL (16.79 ± 4.43 vs. 19.86 ± 4.99) were significantly lower in the LCOS group than in the No-LCOS group (*P* < 0.001 and *P* = 0.015, respectively) (Table 2).

### 3.2. Primary outcome

Regression analysis was performed to further analyze the correlation between echocardiographic parameters and LCOS. The results of the univariate regression analysis showed that the LVEF on the first and second postoperative day was associated with the development of LCOS and that the RVFAC, TDI s’, RVFWSL, and RV4CSL were associated with the development of LCOS. A multivariate regression analysis was performed to further verify the value of each parameter in predicting LCOS. The results of the analysis showed that only RVFWSL remained correlated with LCOS (Table 3).

**TABLE 3:** Univariate and multivariate analysis to predict LCOS.

	*B*	OR (95% CI)	*p*
**Univariate analysis**			
RV-FAC (%)	2.462	11.728 (3.260–42.20)	<0.001
TDI s’ (cm/s)	0.387	1.473 (1.123–1.933)	0.005
TAPSE (mm)	0.098	1.103 (0.998–1.218)	0.055
RVFWSL (%)	0.318	1.374 (1.177–1.604)	<0.001
RV4CSL (%)	0.134	1.143 (1.024–1.276)	0.017
LVEF on the 1st postoperative day (%)	0.055	1.057 (1.010–1.106)	0.016
LVEF on the 2nd postoperative day (%)	0.064	1.066 (1.020–1.114)	0.004
**Multivariate analysis**			
RV-FAC (%)	1.115	3.048 (0.559–16.621)	0.198
TDI s’ (cm/s)	0.001	1.000 (0.729–1.373)	0.999
RVFWSL (%)	0.516	1.676 (1.258–2.232)	<0.001
RV4CSL (%)	–0.294	0.745 (0.589–0.943)	0.014
LVEF on the 1st postoperative day (%)	0.016	1.016 (0.929–1.111)	0.733
LVEF on the 2nd postoperative day (%)	0.028	1.028 (0.946–1.117)	0.516

LCOS, low-cardiac-output syndrome; RV-FAC, right ventricular fractional area change; TDI, tissue Doppler imaging; TAPSE, tricuspid annular plane systolic excursion; RVFWSL, right ventricle free wall longitudinal strain; RV4CSL, right ventricle four-chamber longitudinal strain; LVEF, left ventricular ejection fraction.

**FIGURE 3:**
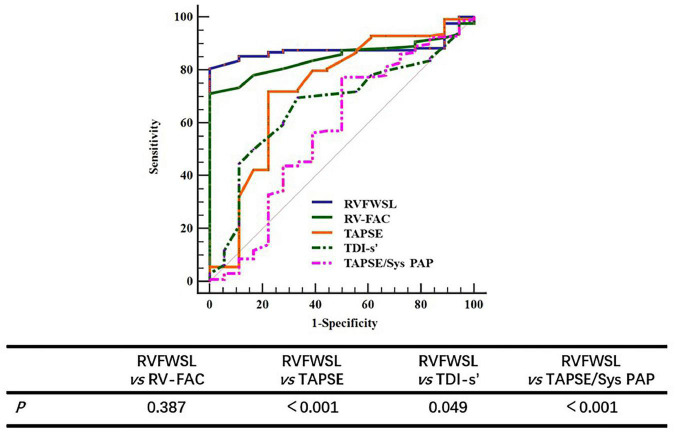
The ROC curve of RV parameters for predicting LCOS. Comparing the value of different parameters in predicting LCOS, the results showed that there was no significant difference between RVFWSL and RV-FAC in predicting LCOS, but there was significant difference between RV RVFWSL and other parameters (*P* < 0.05).

Receiver operating characteristics curve analysis was performed to further determine the diagnostic cut-off value of RVFWSL for predicting LCOS. The results showed that the diagnostic cut-off value for RVFWSL to predict LCOS was less than –18.3, with an area under the curve of 0.879, sensitivity of 100%, and specificity of 80.47% (Figure 3).

In order to further demonstrate that the value of RVFWSL in predicting LCOS is better than other parameters of evaluating right ventricular function, we further analyzed the results of RV-FAC, TDI’s, TAPSE and TAPSE/Sys PAP in predicting LCOS. The results showed that the AUC of FAC was 0.848 (95% CI: 0.780–0.902), the AUC of TDI s’ was 0.728 (95% CI: 0.648–0.798), and the AUC of TAPSE was 0.667 (95% CI: 0.585–0.743), the AUC of TAPSE/Sys PAP was 0.576 (95% CI: 0.491–0.657) (Table 4). The AUC of RV-FAC, TDI’s, TAPSE and TAPSE/Sys PAP in predicting LCOS is less than that of RVFWSL. Comparing the value of different parameters in predicting LCOS, the results showed that there was no significant difference between RVFWSL and RV-FAC in predicting LCOS (*P* = 0.387), but there was significant difference between RV RVFWSL and other parameters (*P* < 0.05) (Figure 3 and Table 4).

**TABLE 4:** The ROC curve of RV parameters to predict LCOS.

	AUC	*P*	95% CI
RVFWSL	0.879	<0.001	0.815–0.927
RV-FAC	0.848	<0.001	0.780–0.902
TDI-s’	0.728	0.002	0.648–0.798
TAPSE	0.667	0.022	0.585–0.743
TAPSE/SysPAP	0.576	0.296	0.491–0.657

LCOS, low-cardiac-output syndrome; RVFWSL, right ventricle free wall longitudinal strain; RV-FAC, right ventricular fractional area change; TDI, tissue Doppler imaging; TAPSE, tricuspid annular plane systolic excursion; Sys PAP, systolic pulmonary artery pressure.

### 3.3. Secondary outcome

The secondary outcome during the follow-up period of this study was readmission of patients within 2 years, including patients readmitted 30 days after their initial admission. Among all patients, 14 were readmitted within 2 years, and all readmissions were for cardiac reasons. There were five readmissions in the LCOS group (incidence of 27.78%) and nine readmissions in the No-LCOS group (incidence of 7.03%), with a statistically significant difference in readmission rates between the two groups (*P* = 0.005) (Table 5).

**TABLE 5:** Re-admission in patients with LCOS.

Variable	LCOS (*n* = 18)	No-LCOS (*n* = 128)	*P*
Within 30 days	0 (0)	4 (14.29)	0.447
Within 2 years	5 (27.78)	9 (7.03)	0.005

LCOS, low-cardiac-output syndrome.

Further analysis of risk factors for readmission within 2 years showed that LCOS was an independent risk factor for readmission, whereas RVFWSL was not significantly associated with readmission within 2 years (Table 6).

**TABLE 6:** Univariate and multivariate analysis to predict re-admission.

	*B*	OR (95% CI)	*P*
**Univariate analysis**			
LCOS	1.626	5.085 (1.480–17.469)	0.010
RV-FAC (%)	1.061	2.890 (0.781–10.704)	0.112
TDI s’ (cm/s)	0.162	1.175 (0.906–1.525)	0.223
RVFWSL (%)	0.015	1.015 (0.930–1.108)	0.793
RV4CSL (%)	–0.007	0.993 (0.889–1.108)	0.897
LVEF on the 1st postoperative day (%)	0.012	1.012 (0.962–1.064)	0.642
LVEF on the 2nd postoperative day (%)	0.021	1.021 (0.974–1.070)	0.389
**Multivariate analysis**			
LCOS	1.693	5.436 (1.109–26.643)	0.037
RV-FAC (%)	0.794	2.212 (0.371–13.182)	0.383
TDI s’ (cm/s)	0.099	1.104 (0.806–1.513)	0.536
RVFWSL (%)	–0.063	0.938 (0.795–1.108)	0.453
RV4CSL (%)	–0.018	0.983 (0.809–1.193)	0.859
LVEF on the 1st postoperative day (%)	–0.003	0.997 (0.912–1.091)	0.953
LVEF on the 2nd postoperative day (%)	0.005	1.005 (0.928–1.089)	0.895

LCOS, low-cardiac-output syndrome; RV-FAC, right ventricular fractional area change; TDI, tissue Doppler imaging; RVFWSL, right ventricle free wall longitudinal strain; RV4CSL, right ventricle four-chamber longitudinal strain; LVEF, left ventricular ejection fraction.

## 4. Discussion

LCOS is the most common and serious complication after SAVR and is strongly associated with short- and long-term postoperative mortality (5). Therefore, it is important to be able to accurately predict the occurrence of LCOS before SAVR. The main finding of this study is that the RVFWSL may be associated with an increased risk for LCOS in patients who undergo SAVR. The occurrence of LCOS after SAVR is a risk factor for readmission within 2 years, whereas the RVFWSL is not.

Possible reasons for the development of LCOS after aortic stenosis and aortic insufficiency are as follows. First, perioperative RV ischemia may be one of the main causes of impaired RV systolic function postoperatively (18). Second, RV volume overload may occur due to tricuspid or pulmonary regurgitation. Third, aortic valve disease may lead to an increase in LV end-diastolic pressure, which leads to pulmonary hypertension and an excessive RV pressure load (9). Finally, a prolonged and sustained increase in LV afterload leads to thickening and fibrosis of the ventricular wall and a decrease in longitudinal function via the septum to the right ventricle (10). These causes directly lead to rapid RV dilatation and an increase in RV end-diastolic pressure (19), which in turn leads to a leftward shift of the septum (20), a reduction in LV preload, and ultimately the manifestation of LCOS. In our study, RV insufficiency parameters, including decreases in the RVFAC, TDI s’, were found to be closely associated with the development of LCOS. And we found that the AUC of RVFAC for predicting LCOS is higher than other parameters. Compared with RVFWSL, there is no significant difference in predicting LCOS, but the AUC of RVFAC is slightly lower than that of RVFWSL. RVFAC shows a better response to RV function, but it is dependent on image quality and requires a complete display of the entire RV structure. However, there was no significant correlation between TAPSE and LCOS in the present study, which is consistent with previous studies. One previous study suggested that TAPSE is a simple measure of longitudinal function but that it may not accurately reflect overall RV function because it is easily measured even with poor image quality, and therefore its reproducibility is poor (13). Additionally, previous studies have revealed contradictions between TAPSE and RVFAC as predictive parameters (21).

After excluding confounding factors, we found that RVFWSL was more closely related to LCOS than traditional parameters of RV insufficiency. Two-dimensional speckle tracking echocardiography is considered a better method for echocardiographic assessment of RV function and has the advantage of showing a more accurate response to RV function than RVFAC or TAPSE (22). Previous studies have found that RV strain parameters have important predictive value for the prognosis of patients with heart failure (23, 24). A recent study also suggested that both RVFWSL and RV4CSL are the most reliable echocardiographic indicators of RV function and that their value in predicting the risk of cardiovascular death exceeds that of RVFAC and TAPSE (13).

The present study showed that RVFWSL had better value than RV4CSL in predicting LCOS. The main fact on which this finding is based is that the septum is an integral part of the left ventricle, and RVFWSL is therefore closer to assessing RV function than RV4CSL. This is consistent with previous views. Cameli et al. (25) also concluded that the RVFWSL has the highest diagnostic accuracy and that it predicts the RV work-per-pulse index.

The secondary outcome in our study was the readmission rate within 2 years. We found that the readmission rate was 27.78% within 2 years for patients who developed postoperative LCOS. The multifactorial regression analysis showed that postoperative LCOS was a risk factor for readmission within 2 years, whereas RVFWSL was not significantly associated with readmission within 2 years. We consider that the main reason for this is related to the small number of patients included in this study.

The present study has some limitations. First, the sample size was small; a study with a larger sample size is needed to confirm the results of this study. Second, the present study did not include patients who died in the perioperative period, so there may be some bias in the population studied. Third, we excluded 13% of patients from the strain analysis because of poor image quality. Indeed, a very important limitation of RV strain remains the reliance on high-quality images and the frame rate.

## 5. Conclusion

Patients with RVFWSL (<18.3%) may be at increased risk for LCOS after SAVR. The occurrence of LCOS after SAVR is a risk factor for readmission within 2 years. RV function monitoring may have some predictive value for the postoperative prognosis in patients undergoing aortic valve surgery.

## Data availability statement

The raw data supporting the conclusions of this article will be made available by the authors, without undue reservation.

## Ethics statement

The studies involving human participants were reviewed and approved by the First Affiliated Hospital of Xi’an Jiaotong University. The patients/participants provided their written informed consent to participate in this study.

## Author contributions

YS and YY conceived and designed the project. Y-JZ and YS interpreted the results and wrote the manuscript. HC, Y-LD, and J-NS collected the data and collated the echocardiographic data. All authors reviewed the manuscript.
